# Supplementary material for: Unbiased Phosphoproteome Mining Reveals New Functional Sites of Metabolite-Derived PTMs Involved in MASLD Development
Source: Int J Mol Sci. 2023 Nov 10;24(22):16172. doi: 10.3390/ijms242216172 (PMC10671570; doi:10.3390/ijms242216172)
Supplement: Supplementary file 1 [file ijms-24-16172-s001.zip › supplementary_Figures.pdf]

Supplementary materials

Table S1: Peptides and proteins identified in mouse liver phosphoproteome by the OS-PTM approach.

Table S2: Peptides and proteins identified in mouse liver phosphoproteome by the CS approach.

Table S3: ∆M peaks distribution per amino acid residue type and per peptide sequence quintile.

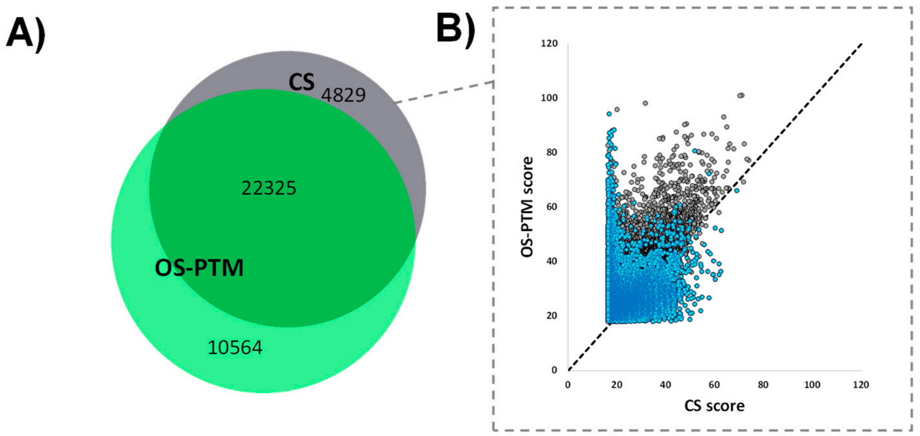

Supplementary Figure 1. Molto et al., 2023

Figure S1. The mouse liver phosphoproteome dataset from the original article[24] was searched with MSFragger in closed search (CS) mode (20 ppm precursor tolerance) using the same variable modifications as in [24] (Met oxidation, Ser, Thr and Tyr phosphorylation, as well as protein N-terminal acetylation), or with MSFragger in OS-PTM mode (from -230 to 1500 Da precursor tolerance). **A**) Area-proportional protein Venn diagrams showing OS-PTM approach confirms most CS plain peptide sequences, but a small percentage containing multiple modifications predefined by the CS with a higher score than in OS-PTM. **B**) The hyperscores obtained for the same spectra in the different searching conditions for the 4829 CS-only peptide sequences are compared. Blue points are PSMs that match peptides with more than one predefined modification in CS mode.

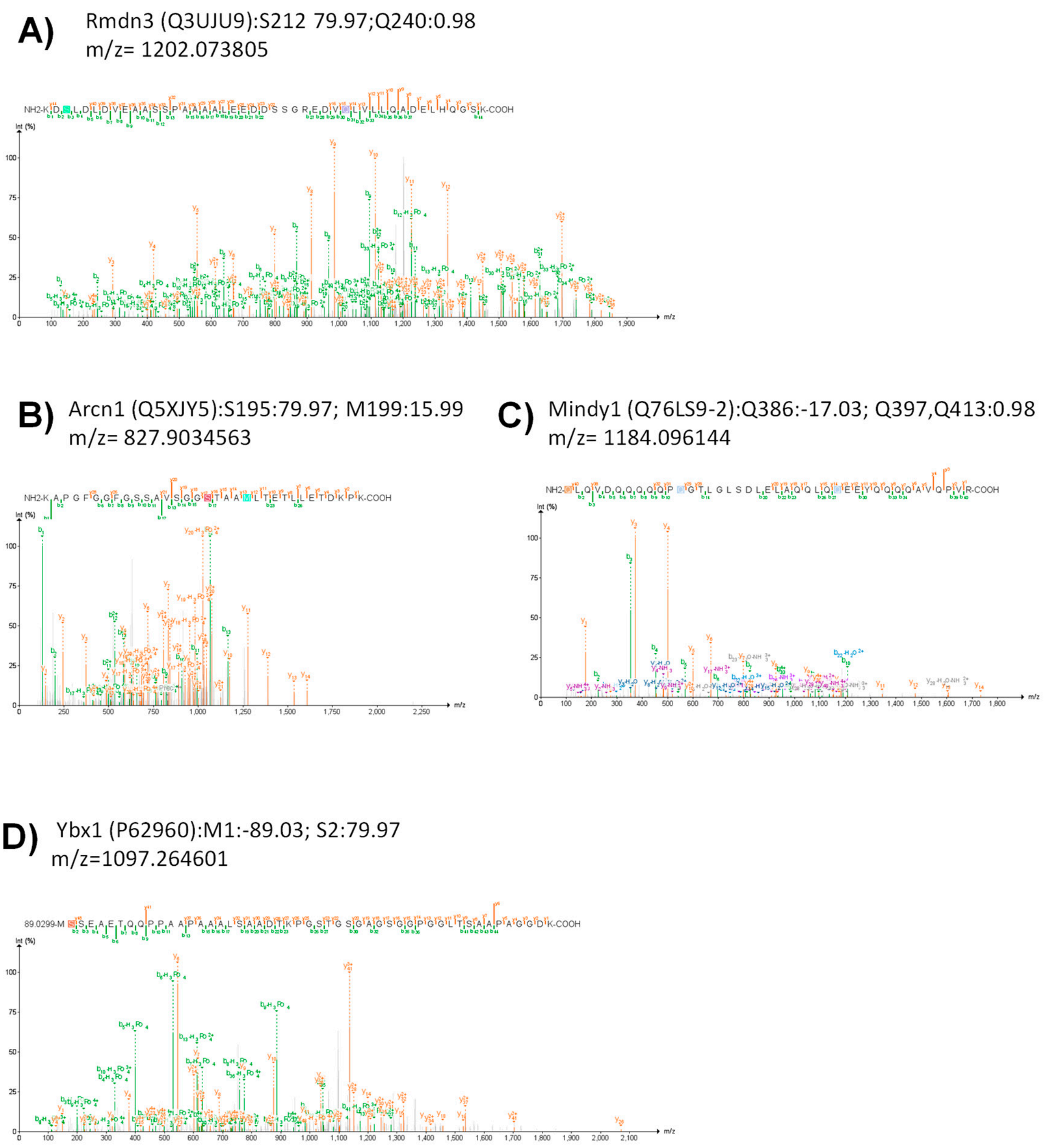

Figure S2: **Identification of peptides modified with unknown ΔM resulting from combinations of abundant known ΔM**. Annotated fragment MS/MS spectra corresponding to (**A**) Rmdn3 ^{210}KD$_{Pho}$SLDLDVEAASSPAAAALEEDDSSGREDV$_{DEA}$QLVLLQADELHQGSK (scan 108325 HFD3_4 raw file), (**B**) Arcn1 peptide ^{179}KAPGFGGFGSSAVSGG$_{Pho}$STAA$_{OX}$MLTETLLETDKPK (scan 98898 HFD3_4 raw file), (**C**) Mindy1 peptide ^{386}$_{AL}$QLQVDQQQQQP$_{DEA}$QGTLGLSDLELAQQLQ$_{DEA}$QEEYQQQQAVQPVR (scan 114529 HFD3_4 raw file), (**D**) Ybx1 peptide ^{1}$_{Mloss+Ac}$M$_{Pho}$SSEAETQQPPAAPAAALSAADTKPGSTGSGAGSGGPGGLTSAAPAGGDK (scan 76297 HFD3_3 raw file). Identified b-ions are marked in green, a-ions in brown, y-ions in red and precursors and immonium ions in grey color. $_{DEA}$Q: deamidated Gln (0.984016 Da); $_{OX}$M: oxidized (15.994915 Da) Met; $_{AL}$Q: ammonia los from peptide N-terminal Gln (-17.026549 Da); $_{Mloss+Ac}$M: removal of initiator Met from protein N-terminus, then acetylation of the new N-terminus (-89.029920 Da); $_{Pho}$S: phosphorylated (79.966331 Da) Ser.

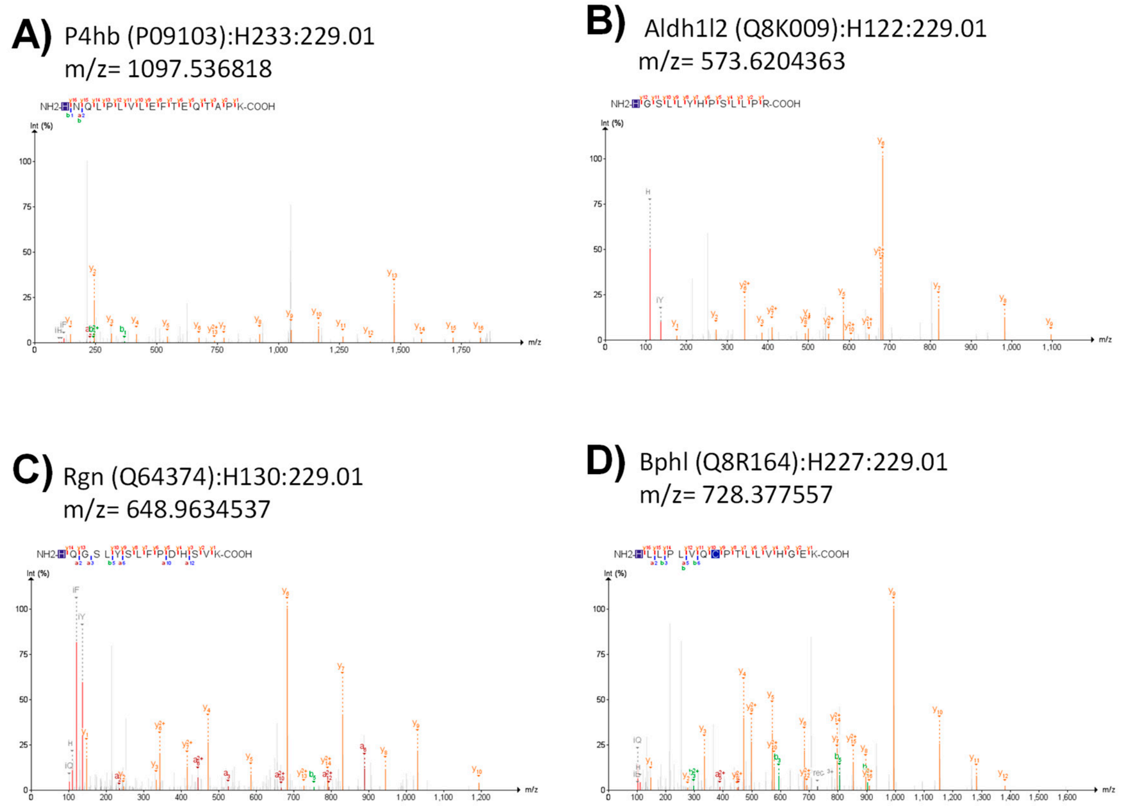

Figure S3**: Identification of peptides modified on N-terminal His with an unknown ΔM similar to pyridoxal phosphate (229.014009 Da).** Annotated fragment MS/MS spectra corresponding to (**A**) P4hb peptide $^{233}{}_{UK}$HNQLPLVLEFTEQTAPK (scan 107832 Control4 raw file), (**B**) Aldh1l2 peptide $^{122}{}_{UK}$HGSLLYHPSLLPR (scan 73824 Control4 raw file), (**C**) Rgn peptide $^{130}{}_{UK}$HQGSLYSLFPDHSVK (scan 84917 Control4 raw file), (**D**) Bphl peptide $^{227}{}_{UK}$HLLPLVQ$_{CAM}$CPTLLVHGEK (scan 104527 Control4 raw file). Identified b-ions are marked in green, a-ions in brown, y-ions in red and precursors and immonium ions in grey color. $_{UK}$H: unknown modification (experimental ΔM=229.0138726 Da) on N-terminal His; $_{CAM}$C: carbamidomethyl (57.021464 Da) Cys.

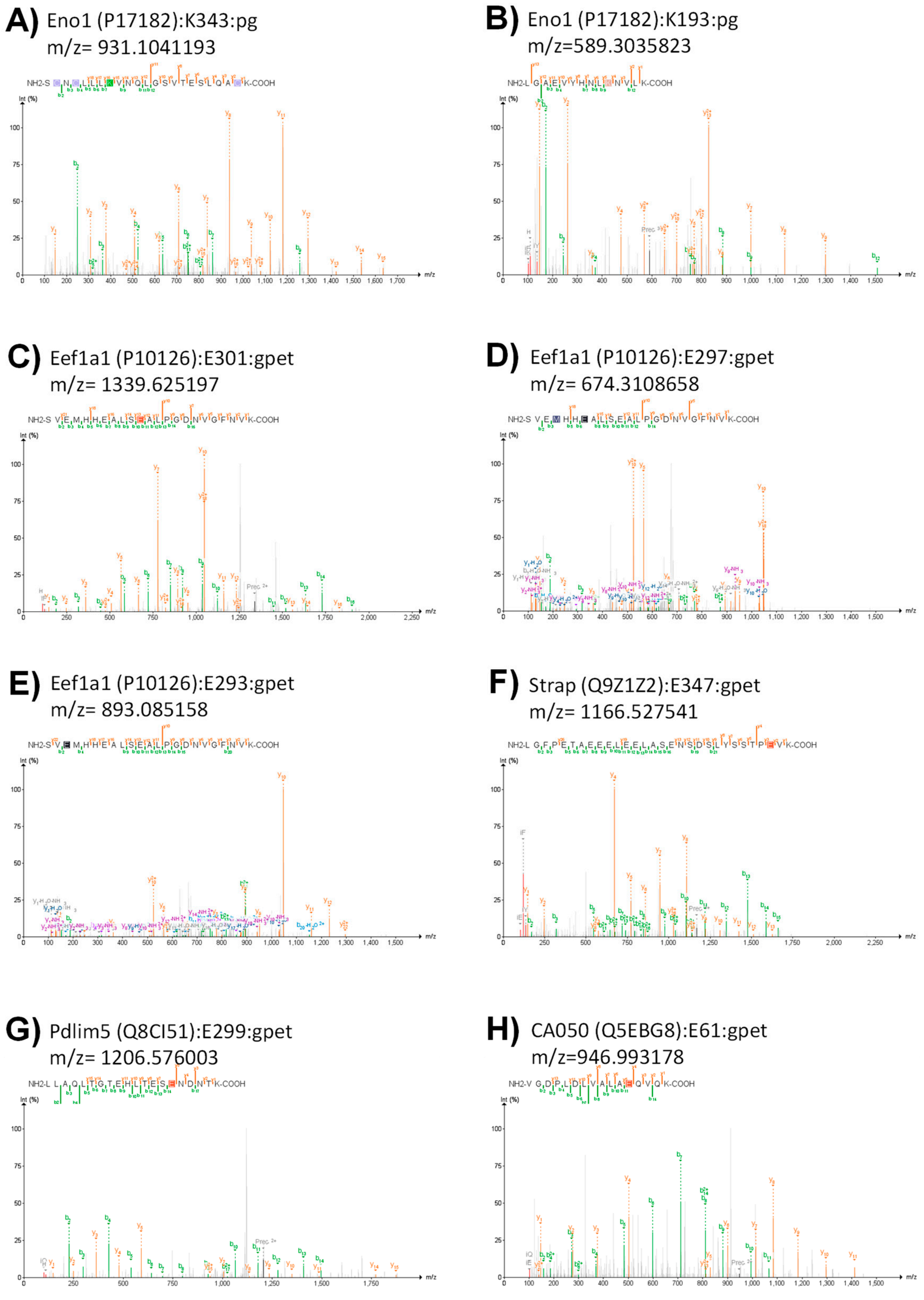

Figure S4: **Identification of Lys-glycerophosphorylated (pgK) and glycerylphosphorylethanolamine-Glu (gpetE) modified peptides**. Annotated fragment MS/MS spectra corresponding to Eno1 peptides (**A**) ^{336}S$_{CAM}$CN$_{CAM}$CLLL$_{pg}$KVNQLGSVTESLQA$_{CAM}$CK (scan 106205 HFD3_4 raw file) and (**B**) ^{184}LGAEVYHNL$_{pg}$KNVLK (scan 82544 HFD3_4 raw file); Eef1a1 peptides (**C**) ^{291}SVEMHHEALS$_{gpet}$EALPGDNVGFNVK (scan 76370 Control4 raw file), (**D**) ^{291}SVE$_{OX}$MHH$_{gpet}$EALSEALPGDNVGFNVK (scan 74550 HFD12_1 raw file) and (**E**) ^{291}SV$_{gpet}$EMHHEALSEALPGDNVGFNVK (scan 60239 HFD3_3 raw file); (**F**) Strap peptide ^{320}LGFPETAEEELEELASENSDSLYSSTP$_{gpet}$EVK (scan 114552 HFD3_3 raw file); (**G**) Pdlim5 peptide

^{285}LLAQLTGTEHLTES$_{gpet}$ENDNTK (scan 58359 HFD12_2 raw file) and (**H**) CA050 peptide ^{50}VGDPLDLVALA$_{GPEt}$EQVQK (scan 110092 HFD12_3 raw file). Identified b-ions are marked in green, a-ions in brown, y-ions in red and precursors and immonium ions in grey color. Pg: 167.982375 Da; gpet: 197.045310 Da; $_{OX}$M: oxidixed (15.994915 Da) Met; $_{CAM}$C: carbamidomethyl (57.021464 Da) Cys.

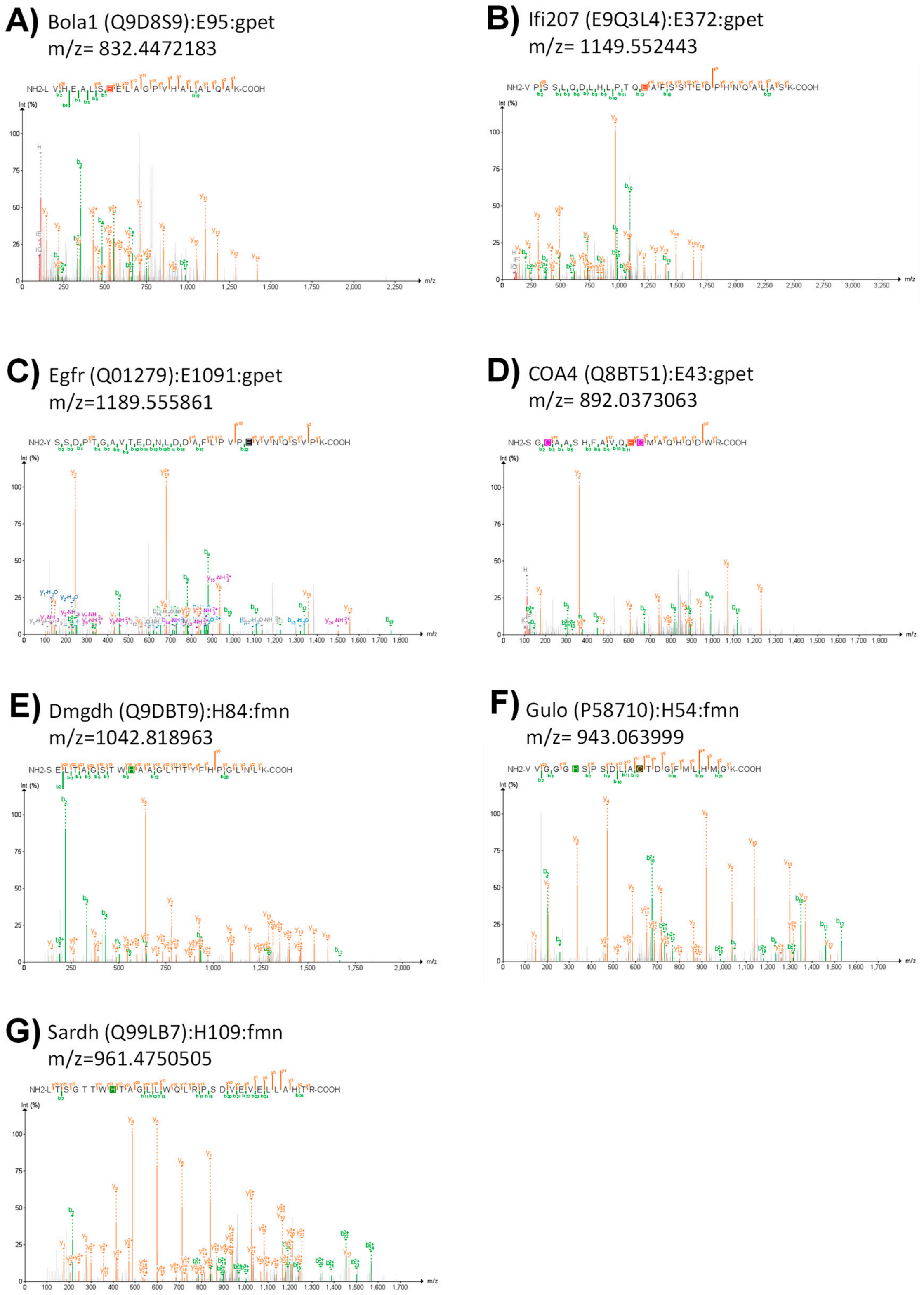

Figure S5: **Identification of glycerylphosphorylethanolamine-Glu (gpetE) and flavin mononucleotide-His (fmnH) modified peptides.** Annotated fragment MS/MS spectra corresponding to (**A**) Bola1 peptide ^{88}LVHEALSE$_{gpet}$ELAGPVHALALQAK (scan 95062 Control_2 raw file); (**B**) Ifi207 peptide ^{359}VPSSLQDLHLPTQ$_{gpet}$EAFSSTEDPHNQALASK (scan 78728 HFD12_4 raw file), (**C**) Egfr peptide ^{1069}YSSDPTGAVTEDNLDDAFLPVP$_{gpet}$EYVNQSVPK (scan 108550 HFD3_4 raw file), (**D**) COA4 peptide ^{32}SG$_{CAM}$CAASHFAVQ$_{gpet}$E$_{CAM}$CMAQHQDWR (scan 56539 HFD12_1 raw file), (**E**) Dmgdh peptide ^{75}SELTAGSTW$_{fmn}$HAAGLTTYFHPGLNLK (scan 92296 HFD12_2 raw file), (**F**) Gulo peptide ^{49}VVGGG$_{fmn}$HSPSDLACTDGFMLHMGK (scan 75580 HFD12_4 raw file), (**G**) Sardh peptide

^{102}LTSGTTW$_{fmn}$HTAGLLWQLRPSDVEVELLAHTR (scan 110891 HFD3_3 raw file). Identified b-ions are marked in green, a-ions in brown, y-ions in red and precursors and immonium ions in grey color. Gpet: 197.045310 Da; fmn: 454.088965 Da; $_{CAM}$C: carbamidomethyl (57.021464 Da) Cys.

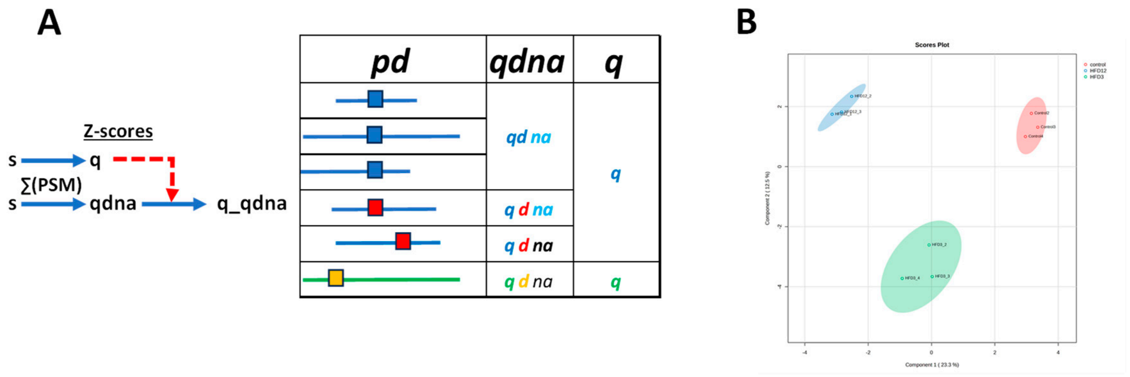

Figure S6: **Quantitative integration workflow for the statistical analysis of label-free OS-PTM experiments.** (**A**) Scheme of the integration workflow used for the quantitative statistical analysis of OS-PTM label-free experiment to increase statistical power to detect differences among conditions. Spectral counts (PSMs) were summed for each element (*q* or *qna*), normalized using total PSM in a particular sample, log2-transformed versus the median from all the samples for the given element and standardized (z-score). Quantitation at the *qdna* level was protein-normalized to detect *qdna* changes independent of their corresponding *q* levels (*Zq_qdna*). Table shows the rules followed to integrate the quantitative information from modified peptides (*pd*) with sequence *p* and delta mass *d*, to protein sites (*qdna*) of protein *q* modified with delta mass *d* on protein residue number *n* and amino acid residue type *a*. (**B**) Principal component analysis (PCA) demonstrating protein quantitation reliability. A reliable set of 3059 proteins quantified in all the samples and for which the PSM sum across the 9 samples was higher than 15 (below that filter were proteins with mainly 1-2 PSM/sample), was selected.

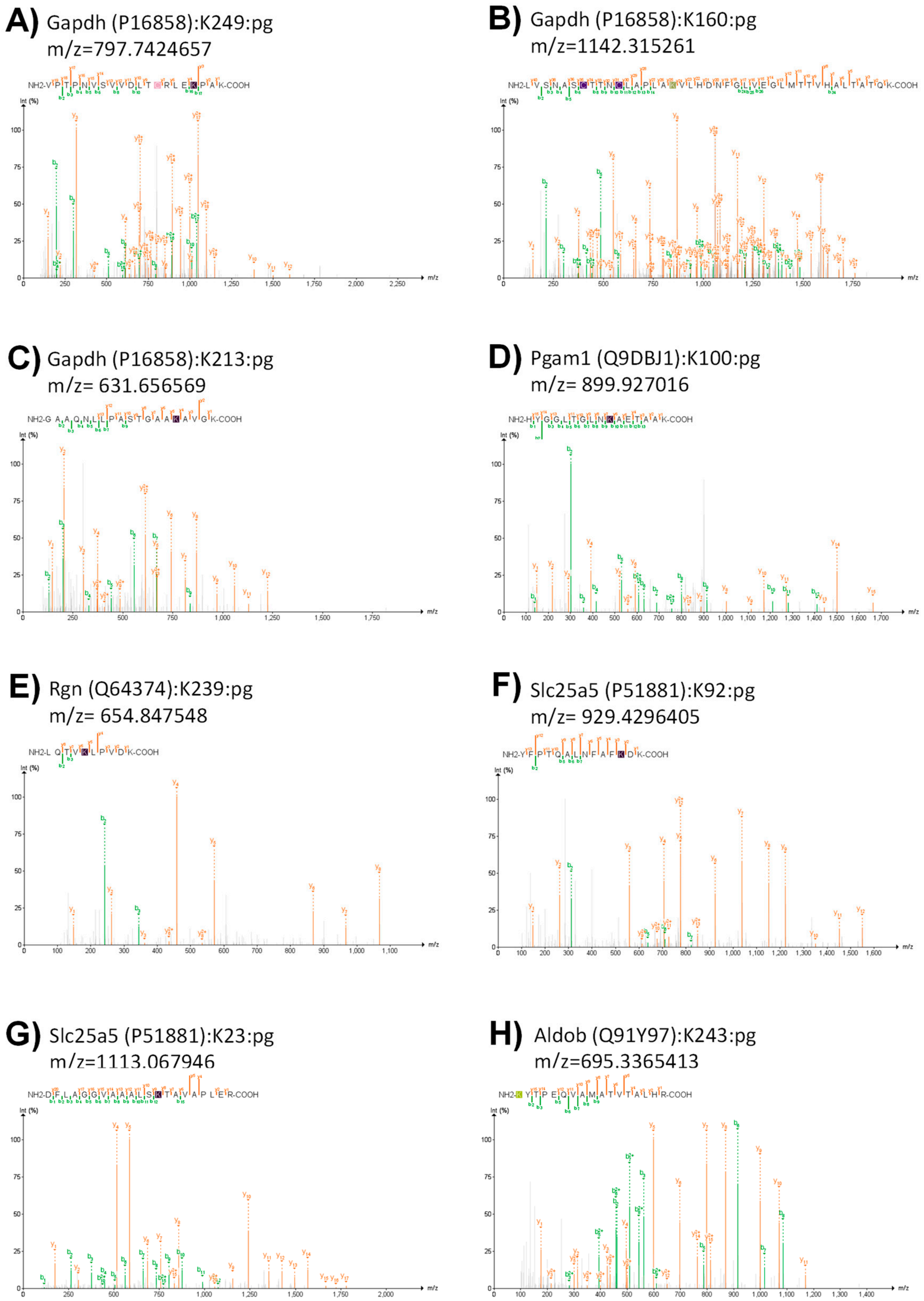
A) Gapdh (P16858):K249:pg
m/z=797.7424657
B) Gapdh (P16858):K160:pg
m/z=1142.315261
C) Gapdh (P16858):K213:pg
m/z= 631.656569
D) Pgam1 (Q9DBJ1):K100:pg
m/z= 899.927016
E) Rgn (Q64374):K239:pg
m/z= 654.847548
F) Slc25a5 (P51881):K92:pg
m/z= 929.4296405
G) Slc25a5 (P51881):K23:pg
m/z=1113.067946
H) Aldob (Q91Y97):K243:pg
m/z=695.3365413

I) Pgls (Q9CQ60):K180:pg
m/z=885.949843

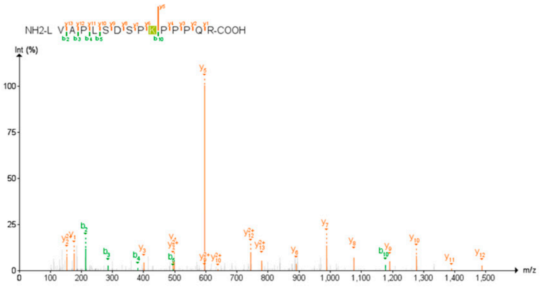

J) Uqcrh (P99028):K83:pg
m/z=474.8815447

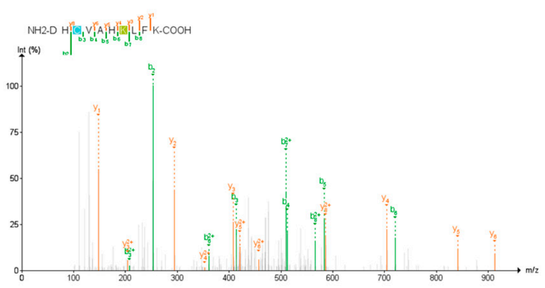

Figure S7: **Identification of the metabolyte-derived phosphate-containing PTMs changing in HFD in mouse liver.** Annotated fragment MS/MS spectra corresponding to Gapdh peptides (**A**) ^{233}VPTPNVSVVDLT$_{CAM}$CRLE$_{pg}$KPAK (scan 89596 HFD3_4 raw file), (**B**) ^{144}LVSNASCTTNCLAPLA$_{pg}$KVLHDNFGLVEGLMTTVHALTATQK (scan 124960 HFD3_3 raw file) and (**C**) ^{199}GAAQNLLPASTGAA$_{pg}$KAVGK (scan 65261 HFD3_4 raw file); (**D**) Pgam1 peptide ^{91}HYGGLTGLN$_{pg}$KAETAAK (scan 51582 HFD3_4 raw file); (**E**) Rgn peptide ^{235}LQTV$_{pg}$KLPVDK (scan 67265 HFD3_4 raw file); (**F**) Slc25a5 peptides ^{81}YFPTQALNFAF$_{pg}$KDK (scan 104126 HFD3_4 raw file) and (**G**) ^{11}DFLAGGVAAALS$_{pg}$KTAVAPLER (scan 114759 HFD3_4 raw file); (**H**) Aldob peptide $^{243}{}_{pg}$KYTPEQVAMATVTALHR (scan 98535 HFD3_4 raw file); (**I**) Pgls peptide ^{171}LVAPLSDSP$_{pg}$KPPPQR (scan 58410 HFD3_4 raw file) and (**J**) Uqcrh peptide ^{77}DH$_{CAM}$CVAH$_{pg}$KLFK (scan 44852 HFD3_4 raw file). Identified b-ions are marked in green, a-ions in brown, y-ions in red and precursors and immonium ions in grey color. pgK: glycerophosphorylated (167.982375 Da) Lys; gpetE: glycerylphosphorylethanolamine-Glu (197.045310 Da); $_{OX}$M: oxidixed (15.994915 Da) Met; $_{CAM}$C: carbamidomethyl (57.021464 Da) Cys.
